# A Preliminary Assessment of Selected Social Determinants of Health in a Sample of Transgender and Gender Nonconforming Individuals in Puerto Rico

**DOI:** 10.1089/trgh.2018.0045

**Published:** 2019-01-30

**Authors:** Jose J. Martinez-Velez, Kyle Melin, Carlos E. Rodriguez-Diaz

**Affiliations:** ^1^ University of Puerto Rico-Medical Sciences Campus, School of Public Health, Center for Sociomedical Research and Evaluation, San Juan, Puerto Rico.; ^2^ University of Puerto Rico-Medical Sciences Campus, School of Pharmacy, Department of Pharmacy Practice, San Juan, Puerto Rico.; ^3^ The George Washington University, Milken Institute School of Public Health, Department of Prevention and Community Health, Washington, District of Columbia.

**Keywords:** Caribbean, gender nonconforming, Puerto Rico, social determinants of health, transgender

## Abstract

**Purpose:** Transgender and gender nonconforming (GNC) people continue to experience suboptimal health care, social exclusion, and lower quality of life. Globally, lack of access to services, institutional violence, and public harassment have been reported. However, there is limited data on transgender health in Puerto Rico and the Caribbean. The purpose of this study is to assess the social determinants of health and wellbeing of transgender and GNC people living in Puerto Rico.

**Methods:** Utilizing a community-based participatory research approach, 52 self-identified transgender and GNC individuals living in Puerto Rico completed a survey, which included questions on access to health care services, social support, and violence, among others. Data were collected from March to Ma y of 2015 and descriptive statistical analysis was conducted.

**Results:** Most of the participants reported experiences of discrimination across multiple social settings, most commonly at school (70.6%) and work (67.4%). Regarding experiences of violence, more than half (65.4%) had been verbally attacked in a public space. Many reported that access to gender-affirming health care services is difficult in Puerto Rico (88.5%) due to lack of knowledgeable providers (59.6%) and discomfort during the encounter (55.8%). The main perceived priority for their wellbeing was a transgender health care center.

**Conclusion:** Although the LGBT equality movement has reached great milestones, access to gender-affirming health services and safe educational and work spaces are still needed. Findings from the study provide guidance for actions to reduce health disparities by addressing the needs for health and wellbeing among transgender and GNC individuals.

## Introduction

Despite the implementation of national and local policies to promote equality and social justice for gender and sexually diverse populations (i.e., same-sex marriage, protection from work discrimination based on sexual orientation and gender identity), transgender and gender nonconforming (GNC) individuals still encounter severe health disparities and challenges for their well-being.^1–3^ Lack of or limited access to appropriate gender-affirming health care, higher rates of violence, public harassment, substance abuse, mental health problems, homelessness, and an increased risk for HIV and other sexually transmitted infections had been repeatedly reported in these groups globally.^4–9^ All of these conditions are influenced by sociostructural factors or social determinants of the health of transgender and GNC individuals.

Consistent with a significant portion of the current literature, we understand transgender and GNC as umbrella terms that people may use if they self-identify as transsexual, queer, agender, bigender, and nonbinary, among others.^10–13^ Whereas, we use the term GNC to refer to people whose gender identity or expression (masculine, feminine, androgyny, among others) is portrayed in a manner different from what is socially and stereotypically expected for men or women at a certain point in our societies.^14,15^

Social determinants of health are described as “conditions in which people are born, live, learn, work, play, and age that affect a wide range of health, functioning, and quality-of-life outcomes.”^16^ Prejudice and stigma toward gender transgressions lead to social exclusion, violence, and experiences of discrimination, which ultimately shape the life course of sexually diverse and GNC individuals. Moreover, the further an individual's gender expression shifts away from stereotypical gender binary constructs, the higher degree of violence, social rejection, and hostility they may face.^17^ These experiences are enacted through family rejection, social marginalization, and institutional discrimination at all levels.^11,18^ As a result, transgender and GNC people are more likely to live in poverty, engage in survival sex work, have an overall lower quality of life, and experience worse health outcomes when compared with other so called sexual minorities, including lesbian, gay, and bisexual individuals.^11,19,20^ This phenomenon has been described as a “slope leading from stigma to sickness.”^21^

Stigma occurs when relatively powerful social groups (i.e., health care professionals, policymakers) devalue marginalized populations because they are perceived as having socially undesirable characteristics.^22,23^ Within the social contexts of Puerto Rico, mainly shaped by religious principles and gender-based perceptions, transgender or GNC individuals have been labeled as deviant, mentally ill, or against nature (God's will).^24^ These prejudices promote a degree of separation between transgender and cisgender people. Such separation enables limited social support, discriminatory actions, and prejudicial attitudes toward transgender and GNC people.^23–26^

Discriminatory acts toward transgender and GNC people extend from their daily lives—family rejection, bullying, noninclusive work environments, housing discrimination—into their health care experiences, encountering multiple barriers in the access to and utilization of health care services.^21,27–30^ Even where transgender and GNC individuals may have access to health care, many receive suboptimal services due to lack of competent health care providers able or willing to meet their health care needs, hostility, and mistreatment in the provision of care, and even refusal of treatment.^27,31^ For these reasons, transgender and GNC people may avoid or delay seeking health care services, which could have a potentially negative impact on their overall health and wellbeing.^8,25,27^

In a recent sample of 27,715 self-identified transgender individuals in the United States, including Puerto Rico, 23% reported that they avoided seeking care due to previous experiences of discrimination, whereas a third (33%) reported having experienced a negative encounter with a health care provider (e.g., refused treatment, verbally harassed, or physically attacked) due to their gender identity.^27^

Historically, local and national surveillance systems have not included standards or reliable questions to collect information about people's sexual orientation and gender identity.^32^ This has created an inaccurate representation of transgender and GNC individuals and their health needs across research and health care services.^33^ Moreover, limited research on transgender health disparities and a lack of systematic collection of information regarding sexual orientation and gender identity are some of the obstacles in the promotion of transgender and GNC people's health and wellbeing.^3,34^ Similarly, transgender health research conducted in Puerto Rico has been limited and primarily focused on HIV risk.^35,36^

More recently, the focus has shifted to also include individual and contextual health risk practices such as silicon use and hormone injections,^37^ and structural factors associated with their wellbeing, including the construction of transgender identities,^38^ transmigration,^39^ violence,^40^ and mental health providers' attitudes, knowledge, and social distance with the transgender community.^41^ Nonetheless, further research is needed and particular attention should be placed on the social and structural factors shaped by the political and economic status of Puerto Rico within the United States.

Considering the needs to further explore sociostructural factors or social determinants of health influencing the health of transgender and GNC individuals in Puerto Rico, a study was conducted to describe the experiences of transgender and GNC people when accessing or receiving health care services and discrimination in multiple social settings such as school, work, and when accessing bathroom facilities. Furthermore, we describe their experiences in relation to gender transition and other health and wellbeing issues.

## Methods and Analysis

Following a community-based participatory research approach previously used by our research team and described elsewhere,^42,43^ we developed a survey instrument to capture the experiences of self-identified transgender and GNC individuals with selected social determinants of health in Puerto Rico. The survey instrument included observations on sociostructural and behavioral aspects reported in the scientific literature, and confirmed by local community stakeholders, as significant factors in determining transgender health such as access to gender-affirming health care, housing, employment, and experiences of violence. For example, items in the survey included questions such as, “Have you ever experienced violence based on your perceived gender identity?” and “Do you consider it's difficult to access to gender affirming health care?” Options to these questions could be answered with yes, no, or don't remember. Demographic data were collected on age, income, relationship status, education, and employment, among others.

To complete the survey, individuals had to self-identify as transgender or GNC, being at least 18 years of age, and living in Puerto Rico at the time of data collection. The data collection instrument was available for self-administration online and on paper format for those with limited access to online platforms. Participants were recruited online and with the support of two local community-based organizations with experience providing HIV services to transgender and GNC populations in Puerto Rico. These organizations shared our recruitment materials on their social media platforms and in their facilities. To facilitate participation, a computer with access to the data collection instruments was made available in our research office for eligible participants.

We were able to recruit 52 participants that completed the data collection instrument using whichever mechanism was more convenient to them. Most participants completed the survey instrument online (*n*=48; 92.3%), one (1.9%) participant went to complete the survey in the computer available in our office, and three (5.8%) completed the paper-based instrument. An assistant member of the research team entered the data collected on paper-based surveys directly into the survey platform. No incentives were offered to the participants. Data were collected from March to May of 2015 and descriptive analyses were performed using SPSS v22. All study procedures for this analysis were approved the University of Puerto Rico Medical Sciences Campus Human Research Subjects Protection Office.

## Findings

Among the 52 individuals who completed the survey, the mean age was 29 years (SD=7.7; range=15–49). As included in Table 1, the majority reported that their sex assigned at birth was male (71.2%) and over a third (36.5%) reported their current gender identity as feminine. Almost a quarter (23%) used local terms, such as “ponka” (twink) and “bucha” (butch) to describe their gender identity. Half (50.0%) of the participants reported their sexual orientation as homosexual (gay or lesbian). Nearly two-thirds (63.5%) of the respondents have initiated a form of transition to represent the gender with which they self-identify, however, 21.2% reported not being interested in such a transition. Over a third (36.5%) reported being currently partnered and 65.4% live in the San Juan metropolitan area.

**Table 1.** Demographic Characteristics of a Sample of Transgender and Gender Nonconforming People in Puerto Rico

Demographic characteristics	Total^a^	Frequency	%
Age, media, SD, range: (15–49)	52	29.0	7.7
Sex assigned at birth	52		
Male		37	71.2
Female		15	28.8
Current gender identity	52		
Feminine		19	36.5
Transgender women		14	26.9
Transformist		13	25.0
Masculine		12	23.1
Genderqueer		9	17.3
Ponka		9	17.3
Transgender men		5	9.6
Bucha		3	5.8
Transvestite		2	3.8
Other		1	1.9
Sexual orientation	52		
Homosexual		26	50.0
Gay		22	84.6
Lesbian		4	15.4
Heterosexual		16	30.8
Bisexual		4	7.7
Other		6	11.5
Age at initiating transition (clothing, physical appearance), range: (13–41)	32	22.0	6.4
Have transitioned	52	33	63.5
Not interested in transitioning		11	21.2
Have not consider transitioning		8	15.4
Currently partnered	52	19	36.5
Live the in San Juan Metropolitan Area	52	34	65.4

^a^ Total number of participants varies as some questions were asked based on participants' characteristics (based on answers to previous questions) and participants had the option of skipping answering questions.

One-third (34.6%) of the respondents indicated that they live alone and 30.8% with their parents or family members. Almost half (40.4%) of the participants reported having completed a college degree and more than half (63.5%) indicated being employed, mostly part-time (48.5%). Furthermore, as included in Table 2, the majority (66.7%) were living under poverty level and almost half (42.3%) reported having engaged in sex work at some point in their lives.

**Table 2.** Selected Social Determinants of Health in a Sample of Transgender and Gender Nonconforming People in Puerto Rico

Selected social determinants of health	Total^a^	Frequency	%
Housing arrangements	52		
Live alone		18	34.6
Live with parents or family members		16	30.8
Live with a partner		11	21.2
Live with friends or roommates		4	7.7
No safe housing		2	3.8
Other		1	1.9
Education attainment	52		
Less than high school diploma		4	7.7
High school		11	21.2
Technical degree		16	30.8
University degree		21	40.4
Bachelor		17	32.7
Master o doctorate		4	7.7
Employment	52		
Employed		33	63.5
Unemployed		19	36.5
Partially employed	33	16	48.5
Have engaged in sex work	52	22	42.3
Poverty	33		
Under poverty level (<$20,000 a year)		22	66.7
Health care	52		
Has health care coverage		45	86.5
Unable to afford health care coverage	7	6	85.7
Has publicly funded health care overage	45	28	62.2
Consider access to health care is difficult	52	46	88.5
Consider that using transgender health care is difficult		46	88.5
Experience of discrimination			
At school setting	51	36	70.6
At work	46	31	67.4
When searching for a job	46	29	63.0
When receiving health care services	51	25	49.0
When receiving government-provided services	47	23	48.9
When using public restrooms	51	23	45.1
When searching for services at court or with the police	48	20	41.7
When buying or renting a house/apartment	43	16	37.2
Experience of violence	52		
In public settings (restaurants, parks, etc.)		34	65.4
From an intimate partner		29	55.8
Based on perceived gender identity		23	44.2
Support transitioning	32		
Have support from family members		21	65.6
Perceived needs for their wellbeing	49		
Trans health care center		31	63.3
Sex/gender change law		29	59.2
Protection for discrimination at work		25	51.0

^a^ Total number of participants varies as some questions were asked based on participants' characteristics (based on answers to previous questions) and participants had the option of skipping answering questions.

Most (86.5%) participants reported having health insurance coverage. Of those, more than half (62.2%) had publicly funded health coverage. Among respondents without health insurance (13.4%), not being able to afford it was reported as the main reason for being uninsured.

Participants were asked if they had endured experiences of discrimination based on their gender identity at different social settings across their lifespan. Almost all participants (98.0%) reported experiences of discrimination, with school being the most common setting where discrimination had been experienced (70.6%). As detailed in Table 2, other settings where participants reported experience of discrimination were at work (67.4%), when searching for a job (63.0%), and when using public restrooms (45.1%).

Regarding experiences of violence, almost half (44.2%) of the participants were mistreated and harassed based on their perceived gender identity. Also, the majority (65.4%) of the participants documented having been verbally attacked at least once in public settings, such as restaurants or parks and more than half (55.8%) had experienced physical, verbal, or sexual violence from an intimate partner. However, more than half (65.6%) of those who had initiated some form of transition expressed support from their family members.

Participants provided information on their perceived needs for the wellbeing of transgender and GNC people in Puerto Rico. Two-thirds of the respondents (63.3%) highlighted the need for access to health services specialized in transgender health care as their main need for their wellbeing. This was followed by laws that allow the gender marker/sex change on the birth certificate and other identification documents (59.2%), and protection for discrimination at work based on gender identity and sexual orientation (51.0%).

Participants were asked to select preferred modalities for receiving comprehensive and gender-affirming health services. The vast majority documented that they would like to have access to mental health care through individual psychotherapy (88.5%), primary medical care (78.8%), and pharmaceutical care (75.0%). As included in Table 3, other preferred modalities for health service delivery documented by this sample were group therapy (67.3%), counseling (61.5%), health education (59.6%), and dental care (59.6%).

**Table 3.** Specific Health Care Practices and Needs in a Sample of Transgender and Gender Nonconfirming People in Puerto Rico

Health care practices and needs	Total^a^	Frequency	%
Preferred modalities for health service delivery	52		
Individual psychotherapy		46	88.5
Primary care		41	78.8
Pharmaceutical care		39	75.0
Group therapy		35	67.3
Counseling		32	61.5
Health education		31	59.6
Dental care		31	59.6
Other		6	11.5
Barriers to health care services	52		
Lack of knowledge of health care providers		31	59.6
Discomfort in health encounters		29	55.8
Discrimination from health care providers		22	42.3
Lack of medical coverage (denied coverage)		22	42.3
Refusal of services (refused treatment)		16	30.8
Inability to pay for services (cannot afford)		14	26.9
Verbal harassment		12	23.1
Physical abuse		2	3.8
Other		4	7.7
Have been asked by a physician about his/her gender identity or sexual orientation	50	27	54.0
Have told his/her gender identity or sexual orientation to a physician	51	40	78.4
Physician has asked about sexual practices	50	26	52.0
Have told a physician about his/her sexual practices	50	27	54.0
Have had negative experiences when sharing gender identity or sexual orientation to a health care professional	47	20	42.6
Hormone use	32		
History of using hormones (current or ever)		20	62.5
Considering using hormones	32	7	21.9
Not interested in using hormones		2	6.3
Cannot afford using hormones		5	15.6
Have used hormones under medical supervision	20	3	15.0
Time using hormones	20		
Less than 2 months		3	15.0
2–6 months		1	5.0
6 months to a year		1	5.0
1–5 years		6	30.0
Over 5 years		9	45.0
Silicone use	32		
Have used injectable silicone		9	28.1
Considering using liquid silicone		3	9.4
Not interested in using silicone		3	9.4
Have used silicone under medical supervision		0	0.0
Gender-affirming surgery	31		
Have had any form of gender-affirming surgery		13	41.9
Have had gender-affirming surgery under medical supervision	13	12	92.3
HIV and other STIs	50		
Have ever been tested for HIV		43	86.0
Have tested for HIV in the last 6 months	43	26	60.5
Have tested for HIV over a year ago		10	23.3
Have not been tested for HIV	7		
Do not know where to go		1	14.3
Not at risk (self-perception)		7	100.0
Fear of result		2	28.6
Previous experience of discrimination when testing		1	14.3
Have been diagnosed with HIV	49	4	8.2
Engaged in HIV care		4	100.0
Have been diagnosed with at least one STI (other than HIV)	49		
Chlamydia		5	10.2
Syphilis		4	8.2
Genital Herpes		3	6.1
Human Papillomavirus		2	4.1
Gonorrhea		1	2.0
Reasons for having received mental health services	49	29	59.2
Depression		19	65.5
Anxiety		19	65.5
Suicidal behaviors		8	27.6
Other		7	24.1
Eating disorders		5	17.2
Substance abuse		4	13.8

^a^ Total number of participants varies as some questions were asked based on participants' characteristics (based on answers to previous questions) and participants had the option of skipping answering questions.

STI, sexually transmitted infection.

Overall, the vast majority (88.5%) considered that the access and utilization of gender-affirming health care services in Puerto Rico is difficult. Respondents documented suboptimal health care services in Puerto Rico due to barriers to access and utilization of those services. Uninformed and noncompetent health care providers regarding issues pertaining to transgender and GNC individuals' health was documented as the main obstacle to health care faced by the participants (59.6%). Discomfort during health encounters (55.8%), lack of medical coverage (42.3%), discrimination from health care providers (42.3%), and refusal of treatment (30.8%) were also reported.

Respondents answered questions about whether they had been asked or had shared their gender identity and sexual orientation with their health care providers. While only half (54.0%) of the participants reported having been asked, more than three-quarters (78.4%) have told their physicians about their gender identity and sexual orientation. Likewise, more than half reported having been asked about their sexual practices (52.0%) and sharing it with their physicians (54.0%). Nearly half (42.6%) reported having had a negative experience when sharing their gender identity or sexual orientation to a health care provider.

Participants were asked if they had ever had, or wanted to have, any gender transition-related procedure. Two-thirds (62.5%) of participants were currently using hormones or had used them and almost a quarter (21.9%) were considering using hormones. Of those who had used hormones, almost half (45.0%) had used them for over 5 years and only 15% had done it under medical supervision. Also, almost a third (28.1%) of the respondents had used injectable silicone and 41.9% had had some gender-affirming surgical procedure, mainly with medical supervision (92.3%).

The majority of the participants (86.0%) reported having been tested for HIV. Of those, two-thirds (60.5%) had been tested in the last 6 months. However, nearly a quarter (23.3%) was last tested over a year ago. Among those participants that had not been tested for HIV (14.0%), low self-perceived HIV risk and fear of the result were documented as the main reasons to avoid HIV testing.

Respondents were asked if they had ever received a diagnosis of HIV and other sexually transmitted infections. Among the sample, 8.2% had been diagnosed with HIV. As detailed in Table 3, participants also reported having been diagnosed with chlamydia (10.2%), syphilis (8.2%), genital herpes (6.1%), human papillomavirus (4.1%), and gonorrhea (2.0%) in their lifetime.

Nearly two-thirds (59.2%) of the participants had received mental health services. Depression and anxiety (65.5%) were reported as the main reasons for looking for such services. Suicidal behaviors (27.6%), eating disorders (17.2%), and substance abuse (13.8%) were also reported.

## Discussion

Consistent with previous studies,^27,44,45^ our preliminary findings describe high prevalence of selected social determinants of negative health outcomes among transgender and GNC individuals. These social determinants include limited access to competent gender-affirming health care services, experiences of violence, structural discrimination, social isolation, economic instability, and adverse health outcomes such as risk for HIV infection and mental health disorders.

From a structural perspective, Puerto Rico's political relationship as a territory of the United States has historically framed the precarious economic and fiscal crisis in the archipelago. As in many other cases, during economic crises managed with austerity, the most socially vulnerable are the most affected.^46–48^ Therefore, analyzing health disparities in Puerto Rico requires acknowledging colonialism as the ultimate social determinant of health. Consequently, the economic and sociopolitical crisis in the territory is a structural barrier to improve health and reduce health disparities among people in Puerto Rico, particularly among otherwise disfranchised populations such as transgender and GNC individuals.

According to the U.S. Census Bureau, 43.5% of the people living in Puerto Rico are under the poverty level.^49^ Our findings suggest that poverty is common among transgender and GNC individuals in Puerto Rico. Of those participants who reported not having health insurance, not being able to afford it was their main reason. Poverty and poor health are inextricably connected,^50^ and transgender people experience more poverty than the general population. This leads to limited access to proper health care services, dangerous work environments (e.g., survival sex work), and lower quality of life.^51^

Most study participants reported pervasive experiences of discrimination in multiple social settings, mainly at school, when requesting health care services, when accessing a public bathroom facility, and at work. These findings are consistent with anecdotal experiences and the scientific literature, which highlight the high levels of discrimination and social marginalization many transgender and GNC individuals endure in every aspect of their lives; from their inner biological family circle to common places such as stores and restaurants.^27,28,52,53^ Such experiences can lead to a never-ending hostile and stressful living scenario that may trigger to mental health disorders such as depression, anxiety, and suicidal thoughts and a lower quality of life.^11,20^ Our participants also reported high rates of physical, verbal, and sexual violence because of being transgender or GNC. Emerging research had shown that transgender individuals are targets of violence on the basis of rejection of gender nonconformity and heteronormativity.^27,54^ In addition, despite a lack of reliable and standardized questions to collect these types of incidents by law enforcement, there have been a staggering number of lethal attacks perpetrated against transgender individuals in the United States.^55^

Access to health care services is fundamental to promote and sustain better health and wellbeing for everyone. However, transgender and GNC individuals commonly experience multiple barriers to access health care that eventually may negatively impact their overall health and could lead to life-threatening situations. Participants in our study confirmed that their access and utilization of health care services in Puerto Rico is difficult. Lack of knowledgeable and competent providers, discomfort during health care encounters, and even refusal of care were reported. Due to these commonly reported experiences, many transgender and GNC individuals may avoid or delay seeking health care services even when sick or injured.^27,56,57^ Furthermore, institutional discrimination and lack of comprehensive transgender care may put their lives at risk by forcing them to seek services in informal settings without proper clinical care and certified professionals (e.g., silicon injections, hormone treatment).^37^ For example, in our study only 15% of participants using hormone treatment were doing so under medical supervision.

We also acknowledge that at the time of data collection, very few clinicians were providing gender-affirming care and most were available only to people with private medical insurance. This is consistent with participants' perception of difficult access to services. Fortunately, local health care services for transgender and GNC individuals have increased from 2017 to 2018, but its impact on access to and quality of care is yet to be assessed.

Social determinants have direct effects in people's health outcomes. Mainly due to pervasive acts of discrimination and stigma, transgender and GNC individuals, particularly transgender women of color, are disproportionally affected by HIV and other sexually transmitted infections.^58^ As reported, almost 10% of our participants reported having been diagnosed with HIV. However, although the majority of the respondents had been tested for HIV at least once, almost a quarter had not been tested in more than a year. This finding is consistent with national data, which highlights that transgender and GNC individuals report a lower prevalence of past year testing and many may not be aware of the HIV status.^59^ Tailored, community-based testing initiatives are needed to encourage transgender and GNC individuals to get tested, reach those at greater HIV risk, and link them to appropriate care.

Participants of this assessment stated, as a main priority for their wellbeing, laws that protect them against discrimination based on their sexual orientation and gender identity. However, at the moment of the data collection, a law against discrimination on employment based on sexual orientation and gender identity was recently approved in Puerto Rico.^60^ This may be an indicator that many people are not aware of the laws that protect them in situations of employment discrimination or otherwise. Lack of knowledge about their legal protections may increase their social vulnerability, weakening their opportunities to access the protection under the law. Greater efforts in increasing public understanding of transgender legal rights should be carried out by the government and other local institutions to secure equal access and justice in cases of discrimination.

Nearly two-thirds of participants (65.6%) shared they had received support from their families during their process of transition. Not surprisingly, a supportive family is a protective factor for mental and physical wellbeing.^61,62^ Social connections–people and institutions—help build resilience among individuals and create resilient communities to better cope with stressful events as rejection and social discrimination.^63^

Findings from this study should be considered with its limitations. Data were collected from a relatively small sample of individuals who self-identified as transgender and GNC recruited with the support of organizations with experience providing HIV services to transgender individuals, mostly transgender women. Therefore, results may be subject to selection bias, and are not necessarily representative of other groups with diverse experiences in Puerto Rico or elsewhere. Consistent with the preliminary and descriptive nature of the study, results and findings are limited on the descriptive data analysis conducted. However, results are consistent with previous similar reports in the scientific literature and should serve as a platform to, with other projects, inform future research and public health actions to address the inequities affecting this population.

## Conclusion

These findings support a call to action for institutions and the government to address the needs for wellbeing and health conditions of transgender and GNC individuals in Puerto Rico. Structural-level initiatives are required to guarantee the implementation of inclusive policies such as gender-affirming health care services, equal protections under the law, and safe educational and work environments for all. The LGBT rights movement has come a long way; however, there is much work to be done to advance the rights of thousands of transgender and GNC individuals. For gender-related social justice and equality to be achieved, addressing the social determinants of transgender and GNC health is a crucial step in halting health inequities.

## Abbreviations Used

GNC: gender nonconforming
STI: sexually transmitted infection
